# Prediction of serious RSV‐related outcomes in older adults with outpatient RSV respiratory illness during 12 consecutive seasons

**DOI:** 10.1111/irv.12751

**Published:** 2020-05-10

**Authors:** Burney A. Kieke, Edward A. Belongia, David L. McClure, Vivek Shinde

**Affiliations:** ^1^ Marshfield Clinic Research Institute Marshfield Wisconsin USA; ^2^ Novavax, Inc Gaithersburg Maryland USA

**Keywords:** adult, penalized maximum likelihood estimation, prediction model, respiratory syncytial viruses, severity

## Abstract

We developed and evaluated a model to predict serious outcomes among 243 adults ≥60 years old with medically attended respiratory illness and laboratory‐confirmed respiratory syncytial virus (RSV); 47 patients had a serious outcome defined as hospital admission, emergency department (ED) visit, or pneumonia diagnosis. The model used logistic regression with penalized maximum likelihood estimation. The reduced penalized model included age ≥ 75 years, ≥1 ED visit in prior year, crackles/rales, tachypnea, wheezing, new/increased sputum, and new/increased dyspnea. The optimal score cutoff yielded sensitivity and specificity of 66.0% and 81.6%. This prediction model provided moderate utility for identifying older adults with elevated risk of complicated RSV illness.

## 1 INTRODUCTION

The epidemiology and burden of respiratory syncytial virus (RSV)‐related respiratory illness is not well‐defined in adults.^1^ The symptoms of RSV mimic those of other viral respiratory pathogens, and specific diagnostic testing is rarely performed in the outpatient setting.^2^ Multiple studies have documented that RSV is an important cause of respiratory illness in adults, particularly those who are immunocompromised or have cardiopulmonary disease.^3^, ^4^ In 2018, we published a report describing the epidemiology and outcomes of RSV infection among adults with outpatient respiratory illness who were systematically recruited and tested for respiratory viruses during 12 influenza seasons.^5^ Among 243 patients ≥ 60 years of age with RT‐PCR‐confirmed RSV, 47 had a serious outcome defined as hospital admission, emergency department visit, or pneumonia. For this study, we analyzed typical clinical and demographic characteristics among patients with RSV in the prior study to develop a predictive model that identifies patients with an elevated risk for a serious outcome as defined above.

## 2 METHODS

### 2.1 Patient population and study design

This was a secondary analysis of a previously published study reporting clinical features, severity, and incidence of RSV illness among older adults living in and around Marshfield, Wisconsin.^5^ Adults ≥60 years old with medically attended acute respiratory illness (primarily outpatient) were systematically recruited by research staff for an influenza vaccine effectiveness study from the 2004‐2005 through 2015‐2016 influenza seasons. Symptom eligibility criteria varied by season, but fever/feverishness or cough were required during most seasons. Multiplex reverse‐transcription polymerase chain reaction (RT‐PCR) was performed on archived samples to detect RSV and other viruses. The multiplex panel included RSV A and B, human rhinovirus, human metapneumovirus, parainfluenza viruses 1‐3, influenza A (H3, H1, and H1N1pdm09), influenza B, and adenoviruses B/E and C. Symptoms were assessed during the enrollment interview, and medical records were abstracted for all RSV cases. Serious RSV outcomes were defined as acute care hospital admission, emergency department (ED) visit for acute illness, or pneumonia occurring within 28 days after enrollment. A physician independently validated all serious outcomes. All participants provided informed consent for influenza study participation, and subsequent multiplex testing was approved by the institutional review board with a waiver of informed consent.

### 2.2 Statistical modeling

The modeling dataset contained information from 243 adults with RSV illness, including 47 who had a serious outcome and 196 with a non‐serious outcome. A model to predict presence of a serious RSV outcome was built via logistic regression using penalized maximum likelihood estimation (PMLE).^6^, ^7^, ^8^ Models employing PMLE, a form of shrinkage estimation, effectively use fewer degrees of freedom (*df*) than standard MLE models and hence have a higher threshold for what would constitute overfitting. In this context, overfitting translates to the number of candidate predictors evaluated being high relative to the number of patients with serious RSV outcome events. PMLE directly adjusts for “over‐optimism,” the fact that predictive performance measures derived from the dataset on which the model was built will generally be more favorable than would be expected when the model is applied to a new data source.

Penalized maximum likelihood estimation is well suited for situations with a high potential for overfitting as described above. However, since the modeling dataset included only 47 serious outcome events, a univariate screening of candidate predictors was performed prior to multivariable modeling. The set of candidate variables included signs and symptoms (both self‐reported and physical examination findings) as well as other clinical and demographic factors.

A goal of the PMLE analyses was to impose shrinkage such that the *df* from the standard MLE‐based logistic regression model (one *df* for each parameter required to represent the terms in the model) were reduced to the point where overfitting was not present. We implemented the shrinkage by selecting a penalty factor and applying this factor in the process of estimating regression coefficients for the full penalized model. Optimization of the corrected Akaike information criterion, a goodness of fit measure, was the basis for selecting the penalty factor. To further reduce the effective *df*, a reduced penalized model containing a parsimonious set of important predictors was established by applying a backward stepdown procedure to the full penalized model.^6^

We assigned a score to each patient that represents the likelihood of a serious RSV outcome using a system based on the estimated regression coefficients. A score of 100 was assigned to the predictor with the largest regression coefficient and the remaining scores reflected the importance of other predictors relative to the predictor with a score of 100. For each patient, scores across predictors were summed to generate a total score.

The final step in the predictive modeling process was to establish an “optimal” cutoff for dichotomizing the score to represent either presence or absence of a serious RSV outcome. We evaluated two well‐known criteria for selecting cutoffs: the “closest to ideal” criterion and the Youden index.^9^, ^10^ In this context, the term “optimal” corresponds to selecting a cutoff with a desirable tradeoff between sensitivity and specificity. We generated an empirical receiver operating characteristic (ROC) curve for the reduced penalized model that depicts the combinations of 1‐specificity (false‐positive rate) and sensitivity (true‐positive rate) across the observed range of possible cutoff values for the patient score. To evaluate the robustness of the selected cutoff, we computed its sensitivity and specificity in 500 bootstrap samples.

Analyses were carried out in TIBCO Spotfire S+® 8.2 for Windows (TIBCO Software Inc), and SAS 9.4 (SAS Institute Inc).

## 3 RESULTS

The univariate screening process yielded 11 variables for inclusion in the initial multivariable model: age ≥75 years, ≥1 ED visit in the prior year, ≥1 hospital admission in the prior year, congestive heart failure (CHF), chronic obstructive pulmonary disease (COPD), examination findings of crackles/rales, fever ≥100°F, tachypnea and wheezing, and patient self‐reported new/increased sputum and new/increased dyspnea. Any coinfection and influenza coinfection status were included in the screening process and neither satisfied the criteria for inclusion in the multivariable modeling. Serious RSV outcomes were more common in patients with coinfections (relative risk = 2.0 overall and 3.2 for influenza coinfections). However, these patients comprised a small percentage of the study population. Only 2.1% (5/243) of patients with RSV had influenza coinfection, and 9.5% (23/243) had coinfection with any virus. The full penalized model yielded 6.5 effective *df*, representing a 41% reduction relative to the corresponding conventional logistic regression model. Area under the ROC curve (AUC) expected in similar patient samples in the future (ie, the AUC corrected for “over‐optimism”) was 0.763. The reduced penalized model accounted for 97.1% of the variation in the full penalized model, used 4.3 effective *df* and contained the following 7 terms: age ≥75, ≥1 ED visit in the prior year, crackles/rales, tachypnea, wheezing, new/increased sputum, and new/increased dyspnea.

**FIGURE 1:**
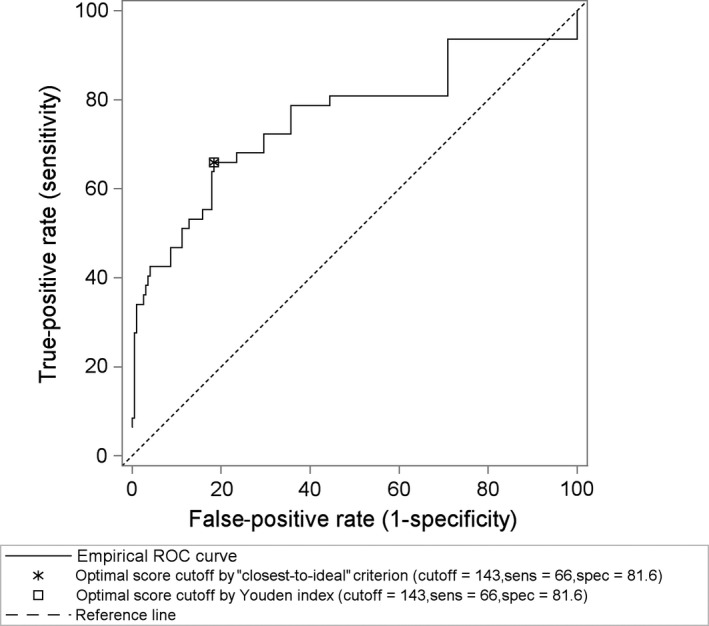
Empirical receiver operating characteristic (ROC) curve for the reduced penalized model

Observed patient scores ranged from 0 to 426. Crackles/rales had the highest score followed closely by tachypnea (Table 1). The “closest to ideal” criterion and Youden index both identified an optimal score cutoff value of 143 with sensitivity and specificity of 66.0% and 81.6%, respectively. The empirical ROC curve for the reduced penalized model, with the selected cutoff annotated, is shown in the Figure 1. In the bootstrap samples, the median sensitivity and specificity were within 0.7% of the observed values. The lower and upper quartiles in the bootstrap samples were within 5.2% of the observed value for sensitivity and 1.8% for specificity**.**

**TABLE 1:** Individual predictor regression coefficients and scores from the reduced penalized model for predicting serious RSV outcomes (hospital admission, ED visit, or pneumonia) among patients ≥ 60 y old with PCR‐confirmed RSV

Predictor	Regression coefficient (RC)	Score^a^
Crackles/rales^b^	0.9651533526	100
Tachypnea^b^	0.9175530877	95
Wheezing^b^	0.6302838213	65
New/increased dyspnea^c^	0.6110782569	63
≥1 ED visit in the prior year	0.5821523979	60
New/increased sputum^c^	0.4156787828	43
Age ≥75 y	0.4133028716	43

^a^ 100 × RC/maximum(RC) rounded to an integer, where the maximum RC is 0.9651533526 from the first row in Table 1.

^b^ Based on examination by a healthcare provider.

^c^ Based on patient self‐report.

## 4 DISCUSSION

This predictive model was moderately successful in predicting serious RSV outcomes in older adults with RSV infection. It was based on patient characteristics readily available in clinical practice and may be useful for identifying and risk stratifying patients with an increased likelihood of serious complications of RSV infection. It could also be useful for constructing clinically meaningful efficacy endpoints for prelicensure clinical trials of RSV vaccines. While the aim of such vaccines would be to prevent serious outcomes such as hospitalizations, ED visits or pneumonia caused by RSV infection, capturing these outcomes typically requires prohibitively large clinical trials. Use of clinical endpoints based on prediction scores derived from readily captured data could be a more efficient approach to measuring vaccine efficacy against serious outcomes of interest.

The strengths of this analysis include systematic screening and testing of patients in the original study, recruitment from a stable community cohort, and inclusion of 12 consecutive seasons with access to outpatient and inpatient electronic medical records. Weaknesses included the small number of serious outcomes captured among adults seeking outpatient care, lack of racial/ethnic diversity, and inability to examine predictors of hospitalization or more severe hospitalized illness. The number of coinfections was small, and it is possible that a larger study would have identified influenza coinfection as a significant predictor of serious outcomes. Validation of this prediction model in larger populations is needed to determine if data collected during an acute respiratory illness visit is useful to predict the likelihood of a subsequent hospital admission or other serious outcome.

In conclusion, a prediction model using clinical information available during the initial evaluation of RSV illness was moderately useful for assessing the risk of a serious outcome.

## CONFLICTS OF INTEREST

EAB, BAK, and DLM have received research support from Novavax, Inc VS is employed by Novavax, Inc
